# Calculating Glycoprotein Similarities From Mass Spectrometric Data

**DOI:** 10.1074/mcp.R120.002223

**Published:** 2021-01-06

**Authors:** William E. Hackett, Joseph Zaia

**Affiliations:** 1 Bioinformatics Program, Boston University, Boston, Massachusetts, USA; 2 Department of Biochemistry, Boston University, Boston, Massachusetts, USA

**Keywords:** Glycoprotein, glycopeptide, similarity, glycosylation, glycoproteomics, bioinformatics, AGP, α1-acid glycoprotein, CCS, collision cross section, DIA, data-independent analysis, ETD, electron transfer dissociation, EThcD, electron transfer higher-energy collisional dissociation, FDR, false discovery rate, HCD, higher-energy collisional dissociation, MRM, multiple reaction monitoring, PRM, parallel reaction monitoring, PTM, posttranslational modification, QTOF, quadrupole time-of-flight, TDA, target decoy analysis

## Abstract

Complex protein glycosylation occurs through biosynthetic steps in the secretory pathway that create macro- and microheterogeneity of structure and function. Required for all life forms, glycosylation diversifies and adapts protein interactions with binding partners that underpin interactions at cell surfaces and pericellular and extracellular environments. Because these biological effects arise from heterogeneity of structure and function, it is necessary to measure their changes as part of the quest to understand nature. Quite often, however, the assumption behind proteomics that posttranslational modifications are discrete additions that can be modeled using the genome as a template does not apply to protein glycosylation. Rather, it is necessary to quantify the glycosylation distribution at each glycosite and to aggregate this information into a population of mature glycoproteins that exist in a given biological system. To date, mass spectrometric methods for assigning singly glycosylated peptides are well-established. But it is necessary to quantify glycosylation heterogeneity accurately in order to gauge the alterations that occur during biological processes. The task is to quantify the glycosylated peptide forms as accurately as possible and then apply appropriate bioinformatics algorithms to the calculation of micro- and macro-similarities. In this review, we summarize current approaches for protein quantification as they apply to this glycoprotein similarity problem.

## Highlights

• Singly glycosylated peptides can be identified unambiguously using HCD LC-MS.
• For calculation of glycoprotein similarity, quantify all glycopeptide glycoforms.
• Glycoprotein similarities can be calculated using the Tanimoto coefficient.
• Similarity calculations require high reproducibility of glycoproteomics LC-MS data.

## Graphical Abstract

**Figure:**
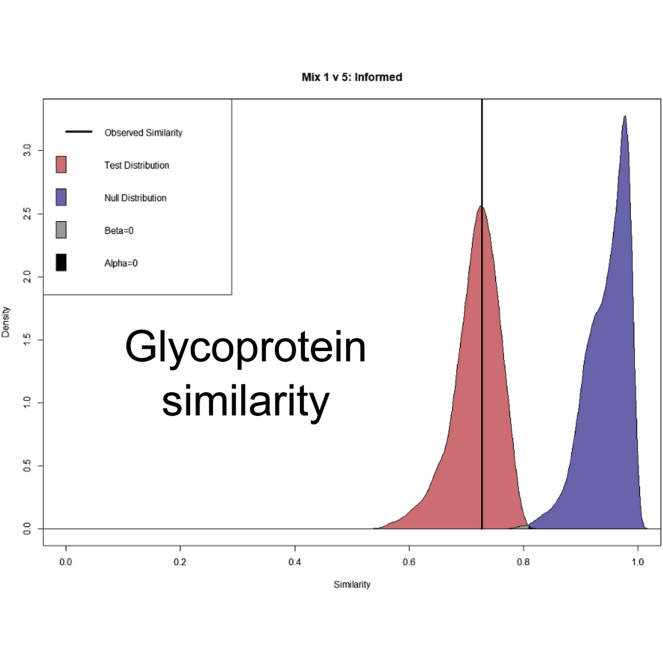

## In Brief

To understand the roles of glycoproteins in biological processes, it is necessary to quantify the changes that occur to glycosylation at individual sites and to the whole molecule. That glycoprotein glycosylation is inherently heterogeneous means that the distribution of glycoforms at each glycosite must be quantified in order to inform calculation of molecular similarities. We review analytical and statistical methods for determining glycoprotein molecular similarities from glycoproteomics data.

## Biological Roles of Protein Glycosylation

Complex cotranslational protein glycosylation in the endoplasmic reticulum serves as a handle for protein folding quality control and sorting in the secretory pathway. The initial glycan cores become elaborated in the Golgi apparatus by biosynthetic enzymes, the functions of which reflect complex balances of substrate concentrations, kinetic, and transport effects. The resulting mature proteins show macro- and micro-heterogeneity of glycosylation that gives rise to populations of mature molecule proteoforms with a distribution of structures and functions (1). Such glycosylation heterogeneity is an evolutionary mechanism whereby protein function with respect to binding to lectin domains is elaborated, giving rise to organized networks at the cell surface and in extracellular matrices through which cells interact with their surroundings, other cells, and pathogens (2, 3).

If the goal of proteomics is to define the flux of protein expression, then that of glycoproteomics is to define the changes to protein site-specific glycosylation that occur in biological processes. That the expression levels of the biosynthetic enzymes, substrate transporters, and core proteins of the secretory pathway are regulated according to cell type, location, development, and disease is well-established. We therefore expect that glycoprotein structure varies spatially and temporally. This is supported by numerous antibody staining and lectin binding studies (4, 5). But there remains a dearth of information regarding how the glycoprotein site-specific glycosylation varies according to the functional requirements of disparate biological systems.

This review describes progress toward the goal of quantifying changes in glycoprotein site-specific glycosylation from bottom-up mass spectral experiments on intact glycopeptides. We place emphasis on analysis of singly glycosylated peptides using label-free collisional dissociation of glycoproteins from biological samples. Recent advances in approaches for glycopeptide quantification, including metabolic labeling approaches, have been reviewed elsewhere (6) and are not described here.

## Assigning Glycopeptides Using Proteomics Methods

Algorithmic assignment of glycopeptides from tandem mass spectra has been covered in recent reviews (7, 8). Briefly, glycopeptides dissociate to produce diagnostic saccharide oxonium ions, neutral losses of monosaccharides from the precursor ion, and peptide backbone product ions. Glycopeptide tandem mass spectra that contain all three types of product ions usually receive the most confident assignments.

With regard to instrumentation, there is a need to distinguish between beam-type collisional dissociation and ion trap resonant collisional dissociation. Beam-type dissociation occurs in triple quadrupole, quadrupole time-of-flight (QTOF), and quadrupole Orbitrap instruments. Multiple collision events between precursor ions and collision gas result in vibrational excitation and dissociation. The extent of dissociation of the precursor ion can be controlled by adjusting the collision energy. Product ions may undergo subsequent dissociation. While beam-type collisional dissociation was used well before the term “higher-energy collisional dissociation (HCD)” was coined, HCD will be used in this review. Resonant collisional dissociation occurs in ion trap instruments whereby a designated precursor ion *m/z* window is collisionally excited. The resulting product ions, having different *m/z* values from the precursor ion, are cooled rapidly by the ion trap bath gas, resulting in a lower extent of dissociation than observed with beam-type dissociation. Ion traps have low mass accuracy relative to QTOF and Orbitrap analyzers. Note that ion trap-Orbitrap instruments can be used to generate either beam-type dissociation (HCD) with high mass accuracy Orbitrap detection or resonant collisional dissociation of precursors with low mass accuracy ion trap detection.

At the present time, high-resolution beam-type collisional dissociation tandem mass spectrometry methods (referred to here as HCD) have been established for assigning the composition of glycosylation existing on a glycopeptide (9, 10, 11, 12). Dissociation of glycosidic bonds occurs more readily than that of the peptide backbone, resulting in a situation where dissociation of the glycan posttranslational modification (PTM) alters the overall product ion pattern. Because traditional peptide database search algorithms and quantification tools do not recognize such glycosidic bond cleavage product ions, specialized tools are required for glycopeptides. In the case of peptides with a single *N*- or *O*-glycan, HCD tandem mass spectra can define the peptide sequence, glycan composition, and site of glycosylation. Often cited practices for producing high-quality HCD tandem mass spectra include enrichment of glycopeptides prior to tandem MS (13, 14, 15) and use of stepped collision energies (10, 11, 16). By contrast, others have used a single high HCD dissociation energy and long LC-gradients to identify coronavirus S-protein glycosylation (17). A number of commercial (18, 19) academic (20, 21, 22) software programs have been cited in recent glycoproteomics publications.

In glycopeptides where more than one *N*- or *O*-glycan is present, glycan dissociation observed in HCD tandem MS often prevents assigning the glycan compositions present at individual glycosites. ETD-based approaches have been used for multiply glycosylated *O*-glycopeptides (23). In addition, β-*O*-GlcNAc modification of Ser and Thr residues is particularly labile and requires special methodological consideration, as described in recent reviews (24). For the purpose of the present discussion, the β-*O*-GlcNAc group dissociates readily during collisional dissociation, making it difficult to assign glycosylated sites (25), and ETD-based methods are therefore preferred (26). Ultraviolet photodissociation also shows great promise for analysis of β-*O*-GlycNAcylated peptides (27).

Electron-activated dissociation methods produce preferential dissociation of glycopeptide peptide backbone bonds (28). Electron transfer dissociation (ETD) is available on many commercial mass spectrometers and, in principle, produces detailed peptide backbone dissociation with much lower extent of glycan dissociation than observed for collisional dissociation. For best results, supplementary activation of ions resulting from ETD is necessary to separate charge loss ions. Available methods include use of supplemental collisional activation (29, 30, 31), often referred to as electron transfer higher-energy collisional dissociation (EThcD), and activated ion ETD (32, 33). Due to the fragmentation to the glycopeptide glycan, collisional dissociation methods determine the overall glycan composition on the peptide. In favorable cases, the site of glycosylation can be assigned from glycosylated peptide backbone product ions. If more than one glycan is present, however, collisional dissociation typically defines the overall glycan composition but cannot differentiate the compositions at individual peptide sites (34). ETD-based methods produce preferential cleavage of the peptide backbone and can be used to assign multiply glycosylated peptides (35). The duty cycle for ETD methods is lower than for HCD due to the ion–ion reaction and supplemental activation times. For this reason, triggering of ETD spectra based on the presence of oxonium ions from HCD spectra is used to conserve analyzer time (29, 36). Because most of the publically available glycoproteomics data sets use HCD methods and define singly glycosylated peptides, we will focus on this aspect in this review.

## Glycopeptide Identification Approaches

The interpretation of glycopeptide tandem mass spectra depends on the completeness of dissociation of the peptide and glycan portions. For collisional dissociation, this depends on the size of the glycan, the peptide sequence, and precursor ion charge state (11). It is important to control the false discovery rate (FDR) for both the peptide and glycan portions of the glycopeptide (16). For this it is necessary to model the extent to which the pattern of peptide+Y ions for a glycopeptide compares with an empirical null model. In order to dissociate as many different glycopeptides as efficiently as possible, researchers have used stepped collision energies (11, 16) at the expense of decreased number of precursor ions selected for tandem MS. As shown in Table 1, there are a number of monosaccharide combinations that can lead to ambiguous tandem MS assignments for glycopeptides.

**Table 1:** Monosaccharide combinations that can lead to ambiguous tandem MS assignments

Saccharide 1	Mass (Da)	Saccharide 2	Mass (Da)	Error (Da)
NeuAc	291.0954	Fuc_2_	292.115	1.02
NeuAc, NH_3_ (adduct)	308.121	Hex, Fuc	308.110	0.011
HexNAc_2_, SO_3_ (substitution)	486.115	Hex_3_	486.158	0.0429
HexNAc_2_, Fuc, NeuAc_2_	1134.407	Hex_7_	1134.369	0.037
NeuAc, Hex	453.148	NeuGc, Fuc	453.148	0.0

In order to augment the evidence in support for a given glycan attached to a peptide, researchers have used chromatographic retention time modeling (20, 37). Using reversed-phase chromatography, it is known that addition of monosaccharide residues to a glycopeptide glycan induces a consistent shift in retention time (29, 30, 31). The retention time shifts have been related to alteration of hydrophobicity of the glycopeptide relative to the bare peptide in the same chromatography system (38). We developed a linear modeling approach for glycopeptides and applied it to two published studies on *N*-glycopeptides (37). We showed that the model was able to correct common errors in glycan assignments due to either insufficient dissociation or ambiguous monosaccharide and adduct combinations. We also determined that glycopeptides produce linear trendlines of collision cross section (CCS) *versus* mass-to-charge ratio (39). These observations support the conclusion that modeling of glycopeptide CCS can be used to augment the assignment of glycopeptides.

To apply target decoy analysis (TDA) (40) to glycopeptides requires generation of an appropriate decoy. A key assumption of TDA is that the scoring pattern of targets and decoys are largely equivalent until the threshold of something being considered a positive or real is reached. If the two sets diverge in scoring well before this threshold, then the assumption is broken and TDA underestimates the FDR. An appropriate decoy for a glycopeptide must produce false scores up to the threshold of reality. The decoy glycopeptides employed in TDA can take several forms. Some softwares, such as GlycReSoft (20), use reversed peptides with identical glycan masses, and no change in glycosite composition; this has the consequence of relying on peptide ions for FDR detection. The pGlyco software (16, 21) creates decoys using mass shifts after generating theoretical fragmentation of reversed peptides and unmodified glycans; its successor, pGlyco2, goes a step further and uses the union of the peptide and glycan FDRs with the glycopeptide FDR subtracted as overlap; this is comprehensive but, like GlycReSoft, runs the risk of removing false glycopeptides since the analysis is composition-agnostic when calculating the overlapping FDR. More complex implementations can help adjust for this, such as permutation to generate new glycans of similar mass; the permuted and nonpermuted glycan decoys can be combined with reversed peptides and unreversed peptides to generate a more comprehensive set of decoys that can be used combinatorially to ensure a more full accounting of false positives is made. This is available in the GlycReSoft multipart search option. GlycoPep Evaluator (41) generates glycopeptides that are isobaric in mass to the target glycopeptides by generating peptides and glycans under a set of rules to roughly match the mass of the targets. This can create a successful set of decoys, but it runs the risk of creating decoys that are in fact true positives instead of false positives. Some softwares (18, 42) fully reverse proteins and find new glycosites and tryptic digests. The challenge is that these can stray too far in mass and general composition to resemble any glycopeptides in the target set. GlycoFragWork (43) combines CID and ETD scoring systems using linear discriminant analysis to generate a single TDA-like evaluation score.

## Glycopeptide Quantification

As described in (44), exploratory proteomics studies using label-free or isobaric labeling result in low to moderate levels of quantification precision. Targeted quantification using multiple reaction monitoring (MRM) or parallel reaction monitoring (PRM) increases the quantitative precision. The most precise relative quantification assays employ a stable isotope-labeled standard for every peptide target. In proteomics, targeted quantification tools including Skyline (45) and PeptideAtlas (46) support the development of methods whereby peptides are quantified using tandem MS product ions. These tools allow PTMs that are chemically defined, including phosphorylation, acetylation, and methylation. As mentioned earlier, the glycan PTM itself undergoes dissociation during tandem MS. Such glycan dissociation, combined with glycosylation heterogeneity, is not presently allowed for quantitative proteomics tools.

**Fig. 1:**
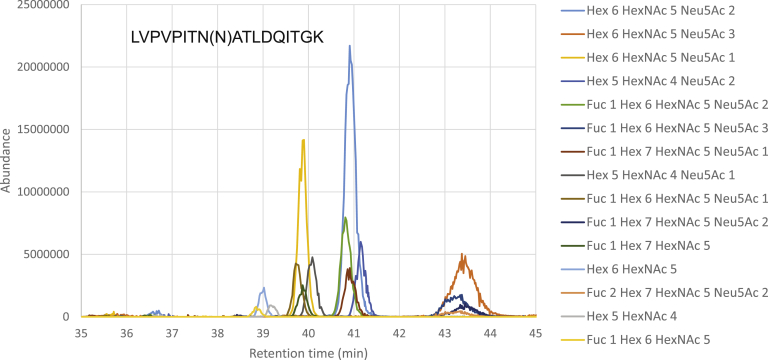
**Example of overlapping AGP glycopeptide 25 to 42 LVPVPITN(N)ATLDQITGK extracted ion chromatograms from published data** (77). The glycosite is given in parenthesis.

Glycopeptide quantification using MS reaction monitoring has been reviewed in detail elsewhere (6). The following discussion is oriented toward the use of MS reaction monitoring for protein similarity measurement. A number of groups have published MRM/PRM methods for quantification of glycopeptides (47, 48, 49, 50). Because peptide backbone dissociation results in low-abundance product ions, the tandem MS transitions often employ neutral saccharide losses from the precursor and oxonium ions. The number of peptides that can be quantified in a targeted MS experiment is limited by analyzer speed. As shown in Figure 1, the heterogeneous glycoforms for a typical glycopeptide modified with complex glycosylation elute from a reversed-phase chromatography column over a narrow retention time window. In order to define glycoprotein similarity, each of the glycoforms observed must be quantified. Such quantification requires 6 to 8 points across the EIC peak. As a result, data-dependent analysis, which selects precursors based on abundance, resulting in stochastic patterns of precursor ion selection, is poorly suited for complete sampling of the heterogeneous glycopeptides. Thus, for many *N*-glycopeptides, there is insufficient analyzer speed to quantify all glycoforms using tandem MS transitions. This pattern of coeluting glycopeptides also complicates the task of building a targeted MS method due to the overlapping EICs, a problem that grows in magnitude as the sample complexity increases.

Data-independent analysis (DIA) methods have been applied widely in proteomics (51). While the advantage is that all precursor ions are dissociated, the challenge to interpretation of DIA proteomics data is to maintain sufficient selectivity of identification of coeluting peptides. Thus, by reducing the size of the quadrupole window used to step through the mass range, the selectivity increases but the scan rate and sensitivity decrease. To interpret DIA tandem mass spectral data, peptide-centric and spectrum-centric approaches have been described (52). Spectrum-centric methods, including DIA-Umpire (53) and OpenSWATH (54), seek to identify a single peptide for each DIA tandem mass spectrum and use a reference library to assign peptides from the DIA data. Peptide-centric methods such as PECAN (55) query the DIA data for the presence of designated peptides using peptide-specific product ions. As such, these methods tolerate the presence of coisolated peptides better than do spectrum-centric approaches.

The presence of many coeluting glycopeptide glycoforms drives the need to design DIA methods with appropriate high selectivity. SWATH-type DIA was used for the analysis of IgG glycoforms, for which heterogeneity is limited, from unfractionated human plasma (56). A DIA method for analysis of biosynthetically truncated mucin-type *O*-glycopeptides has been described in which *in silico* augmented Glyco-DIA spectral libraries were used to assign glycopeptides from unenriched serum (57). A targeted DIA method was used to detect 59 *N*-glycosites from 41 glycoproteins from HILIC-enriched serum for which glycopeptide Y ions were calculated manually and imported into Skyline to generate transitions for quantification (58). Khoo *et al.* noted that while the optimal collision energy for glycopeptide depends on the peptide sequence, the dependence on the glycan composition is small by comparison (59). Thus, they showed that setting collision energy to optimize the abundance of the peptide+HexNAc Y1 ion allows the definition of tandem MS transitions that are specific to the peptide sequence but allow detection of any glycan composition. This approach allows for unanticipated glycoforms to be identified using DIA.

Although there remains no easy way to use existing proteomics DIA analysis software for glycoproteomics, due to the previously mentioned glycosylation heterogeneity and glycosidic bond dissociation, a DIA approach based on profiling the abundances of HexNAc and sialic acid oxonium ions was developed for the purpose of comparing the similarities of multiply glycosylated biotherapeutic glycoproteins. Termed broadband collision-induced dissociation (bbCID), this method was used to assign glycopeptides from standard proteins (60). For this, oxonium ions were used to indicate MS1 scans from which precursors were assigned manually and glycopeptides assigned from the corresponding tandem mass spectra.

### Statistical Analysis Methods for Calculation of Glycoprotein Similarity

In order to assess the roles of glycoprotein glycosylation in biological mechanisms, it is necessary to determine whether a mutated form of a glycoprotein is similar to the wildtype version. This entails combining the abundances of the glycoforms at each site and using a statistical metric to assess the similarity since more traditional statistical analyses are hamstrung by statistical power. In the field of chemoinformatics, molecular similarity refers to similarity of structural or functional molecular properties (61). It is used in drug design studies and in screening chemical structural databases for available compounds with similar chemical properties (62). Molecular similarity is akin to the inverse distance between a pair of compounds in descriptor space. To enable similarity screening of large compound databases, molecules are represented using molecular screens or molecular fingerprints. A number of commercial (63) and public (64) fingerprint databases have been used to screen orphan drug candidates based on similarity. Similarity measures are then used to compare the molecular structures through the fingerprint information. The Tanimoto coefficient (65) is the most often used similarity measure for comparing chemical structures using molecular fingerprints (66). Originally developed to classify plants, the Tanimoto coefficient uses binary presence/absence data to evaluate co-occurrences that reveal relationships among biological or chemical species. The Tanimoto coefficient corresponds to the ratio of the intersection to the union for a species pair. A hypothesis test for organism similarity based on presence/absence data has been developed using the Tanimoto coefficient with bootstrap and measurement concentration algorithms (67).

A glycoprotein contains a set of glycosites, each with a distribution of biosynthetically related glycoforms. A glycoproteomics experiment determines the monosaccharide composition and an abundance for each observed glycopeptide glycoform. The complexities of glycoprotein glycosylation patterns require appropriate statistical metrics for measuring the degree of molecular similarity. We recently developed a modified form of the Tanimoto coefficient to determine statistical similarity between pairs of glycoprotein preparations based on the presences and abundances of glycopeptide glycoforms and applied it to compare wildtype and mutant influenza A virus hemagglutinin glycosylation (68). For this purpose, we used a modified form of the Tanimoto coefficient, shown below:

$$T\;=\;\overset{g}{\underset{i}{\sum }}\frac{A_{i}P_{A,i}B_{i}P_{B,i}K^{-d\left(A_{i},B_{i}\right)}}{\sum_{i}^{g}{\left(A_{i}P_{A,i}\right)}^{2}\;+\;\sum_{i}^{g}{\left(B_{i}P_{B,i}\right)}^{2}\;-\;\sum_{i}^{g}A_{i}P_{A,i}B_{i}P_{B,i}K^{-d\left(A_{i},B_{i}\right)}}$$

**Fig. 2:**
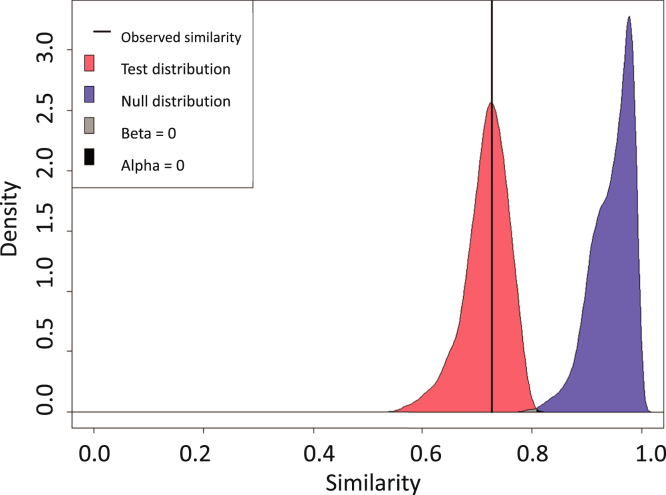
**This example of a similarity comparison shows two glycosites that are differentiable from one another as determined by the low degree of distribution overlap.** These glycosites are the same site on the same protein, but one has worse-quality data than the other due to other glycoproteins injected and processed along with it confounding the signal.

**A** and **B** correspond to the glycopeptide abundance vectors for two glycoprotein samples. These abundances are log-scaled and standardized to yield values in the continuous range of 0 to 1. The glycopeptide abundances are measured in technical repeats that are averaged. The P_A_ and P_B_ vectors contain the proportion of observed values to total values across technical replicates, thereby accounting for missing values. *K* is a distance scaling term $\left[1+mean\left(P_{\text{A}},P_{\text{B}}\right)\right]$, and *d*(A,B) is the Manhattan distance between **A** and **B**. The resulting plot shows two distributions of similarity coefficients, a null hypothesis distribution, and a test hypothesis distribution. The null hypothesis Tanimoto coefficient distribution is calculated from random combinations with replacement of all replicates for samples **A** and **B** in order to simulate a joint distribution. The test hypothesis Tanimoto coefficient is calculated from random combinations of replicates of **A** to those of **B**. As shown for the idealized case in Figure 2, the degree of overlap of null and test distributions determines the confidence with which we can quantify the glycosylation similarity between the sample groups. The x-axis shows the Tanimoto similarity and the y-axis the distribution density. The null and test distributions are analogous to the null and alternative hypotheses used in statistical inference. If the null and test distributions do not overlap significantly, then the null hypothesis is rejected and the samples are dissimilar. If the null hypothesis is not rejected, then the sample pair is not dissimilar. The area to the right of the point of intersection is analogous to the Type I error (α), corresponding to false-positive rate as results in this region could be from the test comparison but have a higher likelihood of being from the null and therefore a negative comparison. The area to the left of the point of intersection is analogous to Type II error (β), corresponding to the false-negative rate. While results in this region could be from the Null comparison, they have a higher likelihood of being from the test comparison and therefore a positive comparison. The plot shows narrow null and test distributions and acceptable levels of Type I and Type II errors.

### Glycopeptide Quantification and Mixture Complexity

Quantitative glycopeptidomics faces challenges that quantitative proteomics does not; focusing on glycoproteoforms intrinsically multiplies ion diversity and reduces quantitation points. Proteomics benefits from the ability to reference multiple peptides when trying to gauge the quantitation of a single proteoform; in contrast, a glycoproteoform can only be evaluated with missed cleavages of the same glycosite. Not only must a complete quantitative assay of a glycoproteome contend with more possibilities than the unglycosylated proteome, but it also must face a decrease in the ability to sample completely the glycopeptide glycoforms to be quantified. To better explain the scale, consider a proteome of 1000 proteoforms. Assuming eight points along the LC elution curve and five peptides per proteoform, this proteome could be quantified by 40,000 successful peptide spectrum matches, 40,000 successful acquisitions. Next, consider the related glycoproteome: based on 20 glycoforms per glycosite, an average of 50% of proteins glycosylated, and just two glycosites per glycosylated protein, there are an expected 20,000 glycoproteoforms. In order to uphold a similar standard of eight points along an elution curve for full quantitation, there needs to be 160,000 successful glycopeptide spectrum matches, 160,000 successful acquisitions. This estimation of a fourfold increase in the sampling power necessary to quantify the glycoproteome is likely to underestimate the multiplicative power of glycosylation because many glycoproteins have more than two glycosites.

The likelihood of precursor ion coisolation must also be taken into consideration in both DDA and DIA experiments. Coisolation of two glycopeptide precursor ions is a more demanding problem than coisolation of two unmodified peptides. In many cases, the peptides can still be identified, assuming that their sequences are not related, but even that is not a certainty. Glycopeptides with a common peptide sequence, however, show a narrow range of LC retention times for the set of glycoforms for a given peptide sequence in *m/z* space; when these glycopeptides are coisolated and dissociated, many of the same peptide+Y_n_ ions and oxonium ions are observed for both precursors. Precursor ion cation adducts, if present, increase the computational work needed to identify the glycopeptides from one another. As shown in Table 1 (37), there are several common combinations of monosaccharides and adducts that lead to precursor ions masses that may lead to false identifications. In the case when two glycopeptides with unrelated peptide sequences coelute, the fact that product ions from peptide backbone dissociation are often low in abundances can result in peak misidentification.

**Fig. 3:**
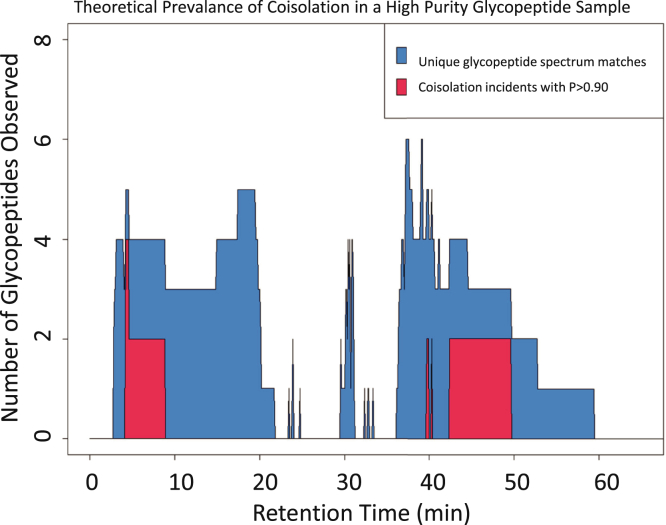
**This shows the number of glycopeptides observed in a purified glycoprotein sample (*blue*) and the number of coisolation events (*red*) that would be likely (*p* > 0.90) given a 10 u window size.** The plots are likely skewed downward due to coisolation events reducing the number of glycopeptide observations.

Given that the ability to sample glycopeptide glycoforms using is limited by analyzer speed, DIA methods are of interest for quantifying glycoprotein glycosites. For such DIA studies, it is important to estimate the probability of multiple precursor ions in the scan window. As a conservative example of this prevalence, we examined a previously published data set examining a purified α1-acid glycoprotein (AGP) sample (69). We created a simple model to estimate the degree of coisolation expected from an experiment with 10 u windows. This model is based on 23 total glycan compositions at five different glycosites in AGP. Based on the analytical data, some sites had as few as three glycan compositions and some with as many as 20, for a total of 60 glycopeptides observed over a 65 min gradient. Figure 3 shows the number of glycopeptides observed at a given time and the number of glycopeptides that had a probability of coisolating greater than 90%. The probability was determined based on window size, mass error of 10 ppm, positive charge states from 2 to 5, and observed time windows of glycopeptides; it was assumed that if a glycopeptide was above a signal threshold, it would produce a tandem mass spectrum. The glycopeptide spectrum matches used to generate these data come from a GlycReSoft search from published data (69), and they should bias away from glycopeptides that coisolate as the search is not set up to handle such instances, so any glycopeptides that do present as likely to coisolate are likely to be edge cases. Overall 12 glycopeptides out of 60 would be expected to be present in the same 10 u window or 20% of identified glycopeptides. This degree of susceptibility indicates the need to differentiate glycopeptides present in the same DIA window. As the glycopeptide mixture complexity increases, the search space expands, and the need to differentiate glycopeptides in the same DIA window increases.

### Quality Standards and Reproducibility

The assessment of changes in glycoprotein glycosylation in biological processes requires careful attention to LC-MS reproducibility. While various quality assessment tools exist, none are standard. Many of the tools that glycoproteomic researchers use are repurposed proteomic tools or expansions on general mass spectrometry tools. While these tools are well-designed and have their own merits, they all lack the complete view needed to assess a glycoproteomic experiment; they are not designed to do so. Glycoproteomics requires an increase in standards from proteomics due to the potential for error and requires specific considerations to handle the idiosyncrasies of its identification processes and quantifications. In regard to FDR, TDA (40) is ill-suited to glycopeptide identification as it relies on the assumption that Decoys mimic targets closely enough to genuinely be confused for Targets by the scoring algorithm. As our ability to score and identify glycopeptides improves, the reliability of our FDR estimation *via* TDA decreases. Various different software have attempted to solve the glycopeptide decoy problem, as discussed above, but none have found a perfect solution as of yet. And while Posterior Error Probability has advanced in recent years (70, 71, 72, 73), it has yet to be implemented, tested, and standardized for glycoproteomics, and many approaches rely on system knowledge that is currently infeasible.

QuaMeter is a quality metric that provides a vast array of metrics regarding the underlying spectra in an experiment and can be applied to any compound class (74). QuaMeter’s assessment of tandem MS coverage, intensity mapping, and noise assessment are useful for performing consistent experiments and adjusting for batch effects (74). The metrics it produces can be compared with benchmarks for the purpose of gauging experimental consistency but do not give insight to identification and quantitation quality.

The Skyline (45) quality tools focus on peptide quantitation. While using Skyline for glycopeptide quantification is possible, it must be carefully implemented to prevent signal contamination between separate glycoproteoforms. At present, glycopeptides and their fragments must be individually added and identified, which makes analysis of a glycopeptide data set time-consuming, especially if an experiment is examining more complex mixtures such as whole tissue. Skyline has an intuitive quantitation assessment tool that uses visualizations to allow the user to ensure that peak boundaries are correct and visual displays to ensure within sample group consistency (29). Glycopeptides need to be treated as ordinary PTMs in MaxQuant, and MaxQuant does not allow for fragment PTMs to contribute to one single identification and therefore quantitation (75, 76). This leads to false-positive identifications as mass shifts are left unaccounted for. MaxQuant can ensure high-quality identifications in the proteomics data used to generate a glycoproteomic search space, but at present cannot be applied directly to glycoproteomics data, meaning that its probabilistic FDR system cannot be applied to glycopeptide identifications.

## Conclusions

Rigorous and complete quantification of glycopeptide data is necessary for making the biomedical discoveries required to meet emerging human health problems. Researchers need tools to properly assess the confidence of glycoproteomics results. This will require development of specific tools and standardization to increase interoperability between experiments. Spectral quality tools, while important, do not paint the whole picture. They lack the perspective of how variation in spectral quality influences interpretation of identification and quantification of glycopeptides. A degradation of signal at a specific time in the elution could lead to a vast shift in results leading to spurious conclusions. To address this problem will require development of holistic tools that inform the user of glycopeptides that are likely candidates for error based on spectral information, search space data, and related sample information. While it is possible for researchers to investigate all of these avenues on their own, the complexity and scope of this task are bound to cause error and are not conducive to generating a set of standards.

Looking to the future, there is a golden opportunity to increase standardization for glycoproteomics. All glycoproteomic experiments must have a known search space. Between this known search space and the ability to predict the expected retention time shifts caused by glycosylation, it should be possible to create a comprehensive data quality analysis. This information can be used to create a glycopeptide coisolation likelihood model as well as predict likely candidates for mass-shifting adducts that closely mimic glycan mass shifts. These two models, in conjunction with spectral data, will determine glycopeptides more likely to produce quantification error. The previously described statistical similarity methods allow for comprehensive examination within an experimental group and can identify problematic glycopeptides and samples as a whole. By using all of these methods together, a researcher can build a consensus on the reliability of their glycopeptide quantification data.

## Conflicts of interest

The authors declare no competing interests.
